# Understanding healthcare provider preferences for ordering respiratory cultures to diagnose ventilator associated pneumonia: A discrete choice experiment

**DOI:** 10.1017/ash.2022.267

**Published:** 2022-07-15

**Authors:** Blaine Kenaa, Nathan N. O’Hara, Lyndsay M. O’Hara, Kimberly C. Claeys, Surbhi Leekha

**Affiliations:** 1 Division of Pulmonary and Critical Care, Department of Medicine, University of Maryland School of Medicine, Baltimore, Maryland; 2 Department of Orthopaedics, University of Maryland School of Medicine, Baltimore, Maryland; 3 Department of Epidemiology and Public Health, University of Maryland School of Medicine, Baltimore, Maryland; 4 Department of Pharmacy Practice and Science, University of Maryland School of Pharmacy, Baltimore, Maryland

## Abstract

**Objective:**

Ventilator-associated pneumonia (VAP) can be overdiagnosed on the basis of positive respiratory cultures in the absence of clinical findings of pneumonia. We determined the perceived diagnostic importance of 6 clinical attributes in ordering a respiratory culture to identify opportunities for diagnostic stewardship.

**Design:**

A discrete choice experiment presented participants with a vignette consisting of the same “stem” plus variations in 6 clinical attributes associated with VAP: chest imaging, oxygenation, sputum, temperature, white blood cell count, and blood pressure. Each attribute had 3–4 levels, resulting in 32 total scenarios. Participants indicated whether they would order a respiratory culture, and if yes, whether they preferred the bronchoalveolar lavage or endotracheal aspirate sample-collection method. We calculated diagnostic utility of attribute levels and relative importance of each attribute.

**Setting and participants:**

The survey was administered electronically to critical-care clinicians via a Qualtrics survey at a tertiary-care academic center in the United States.

**Results:**

In total, 59 respondents completed the survey. New radiograph opacity (utility, 1.15; 95% confidence interval [CI], 0.99–1.3), hypotension (utility, 0.88; 95% CI, 0.74–1.03), fever (utility, 0.76; 95% CI, 0.62–0.91) and copious sputum (utility, 0.75; 95% CI, 0.60–0.90) had the greatest perceived diagnostic value that favored ordering a respiratory culture. Radiograph changes (23%) and temperature (20%) had the highest relative importance. New opacity (utility, 0.35; 95% CI, 0.17–0.52) and persistent opacity on radiograph (utility, 0.32; 95% CI, 0.05–0.59) had the greatest value favoring bronchoalveolar lavage over endotracheal aspirate.

**Conclusion:**

Perceived high diagnostic value of fever and hypotension suggest that sepsis vigilance may drive respiratory culturing and play a role in VAP overdiagnosis.

Ventilator-associated pneumonia (VAP) remains one of the most common hospital-acquired infections and is associated with high morbidity and mortality.^1,2^ Diagnostic workup for VAP should be initiated when chest imaging findings are supported by changes in temperature, white blood cell count (WBC), sputum characteristics, and worsening oxygenation requirements.^3^ However, those findings can be nonspecific and misleading, especially in the presence of cardiac and pulmonary comorbidities.^4,5^ Combined with a low threshold for lower respiratory tract culturing, this makes VAP a frequently overdiagnosed condition.

In our previous study that examined healthcare provider (HCP) perceptions of VAP diagnosis and treatment, most HCPs considered clinical changes or attributes, such as increase in oxygen requirement, temperature and sputum characteristics, and radiographic changes to initiate diagnostic work-up.^6^ However, HCPs also described a low threshold for culturing due to a fear of missing a diagnosis.^6^ Specifically, it is unclear whether respiratory-specific changes, such as radiographic changes, worsening oxygenation, and sputum characteristics, are considered equally by clinicians as nonspecific clinical characteristics, such as changes in temperature, WBC, and blood pressure. The purpose of this study was to quantify the extent to which those clinical changes influence an HCP’s decision to perform respiratory culturing in intensive care unit (ICU) patients. We hypothesized that nonspecific clinical changes or diagnostic criteria were as likely to drive culturing as respiratory-specific attributes and thus might provide motivation for diagnostic stewardship interventions at the time of respiratory culture ordering. Furthermore, in our prior study, HCP in our institution demonstrated a preference for invasive respiratory sampling using bronchoscopy with bronchoalveolar lavage (BAL) or mini-BAL over blind endotracheal sampling (ETA) for VAP diagnosis.^6^ Therefore, as a secondary objective, we estimated the influence of these clinical changes on requests for invasive sampling (ie, BAL) versus noninvasive ETA.

## Methods

### Study design

The study used a discrete choice experiment (DCE) to elicit the perceived diagnostic value of clinical changes associated with VAP diagnosis and included a cross-sectional survey of HCP involved in the care of patients with suspected VAP. The DCE method is a well-established econometric approach, and this study design is increasingly being applied to healthcare research.^7^ DCEs assess individual preferences by presenting participants with multiple hypothetical scenarios and asking them to select their preferred option for each scenario. The underlying assumption in a DCE is that the respondents’ choices are based on maximizing their perceived diagnostic value or utility. Through the systematic construction of response options and analysis of respondents’ choices, researchers can assess the relative importance or diagnostic value of each attribute to the respondent group. Response options are based on a fixed set of attributes of interest and corresponding levels of those attributes; the combination of attributes and levels varies in each scenario.

**Fig. 1.**
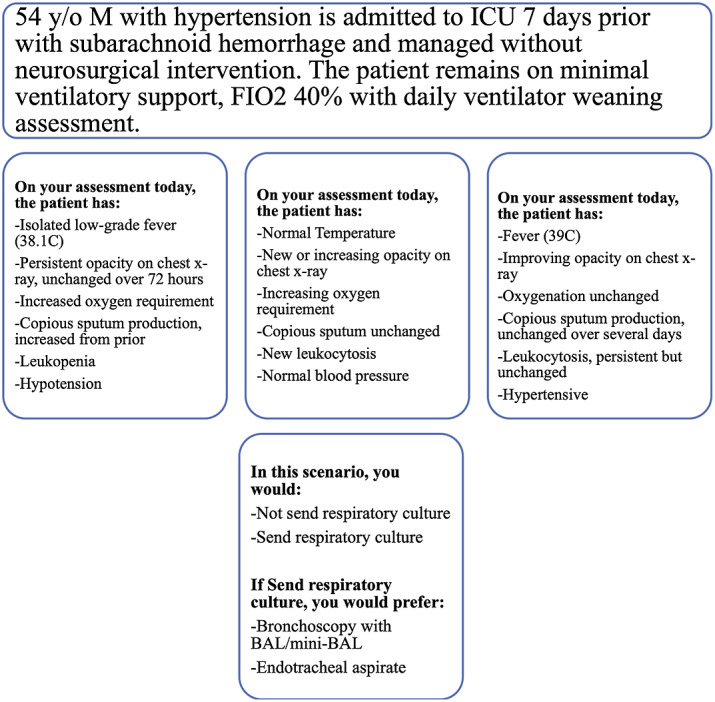
Case vignette with examples of plausible scenarios.

In our study, attributes were defined as clinical changes associated with a diagnosis of VAP. These attributes were informed by clinical guidelines for diagnosis and management of VAP,^2,3^ our previous literature review,^8^ and a qualitative study^6^ eliciting clinicians’ approach to VAP diagnosis. In total, 6 attributes were identified as key for VAP diagnosis: 3 respiratory-tract–specific attributes (ie, chest imaging findings, oxygenation or respiratory status, sputum quantity) and 3 systemic, nonspecific attributes (ie, temperature, WBC, and blood pressure). To develop the 32 clinical scenarios for the survey, each attribute of interest was further defined in to a range of corresponding levels. For example, chest imaging levels included (1) new or increasing opacity, (2) improving opacity, (3) no opacity, and (4) persistent opacity, unchanged over the previous 72 hours. Blood pressure levels were defined as (1) hypotension, (2) unchanged from baseline, (3) hypertensive, or (4) normal. In the survey, respondents were presented with 32 clinical scenarios in which the patient’s condition was described using the same “stem” with a range of plausible attribute levels (Fig. 1). The respondent selected whether or not they would order a respiratory culture, given the described clinical changes. If the respondent indicated that they would order a respiratory culture, they were then asked to indicate whether they would request bronchoscopy with BAL or mini-BAL versus ETA. Given that each of the 6 attributes had 3–4 levels, there were 3,072 possible scenario combinations. The experimental design was created using JMP Pro version 14 software (SAS Institute, Cary, NC) with a D-efficiency^9^ approach to maximize the orthogonality within the 32 scenarios. A complete list of attributes and corresponding levels as well as the survey questionnaire is provided in the Supplementary Material. The study was determined to be non–human-subjects research by the Institutional Review Board of the University of Maryland, Baltimore (no. HP-00083377).

### Study setting and participants

This study was conducted at an urban, academic, tertiary-care hospital. With the goal of including those directly or indirectly involved in diagnosis and treatment of patients with suspected VAP, HCP invited to participate in the survey included ICU attending physicians, clinical fellows, residents, advanced practice practioners (nurse practitioners or physician assistants), and clinical pharmacists.

### Survey administration

The survey was constructed using the Qualtrics online survey platform (Qualtrics, Provo, UT) and consisted of the 32 clinical scenarios plus basic demographic questions of the respondents. Invitation to participate in the survey was sent via e-mail between September 16, 2020, and October 23, 2020. Up to 3 reminders were sent to eligible participants.

### Statistical analyses

Response data were analyzed using mixed probit models (R version 4.0.0 software, R Foundation for Statistical Computing, Vienna, Austria). The attribute-level coefficients are referred to as the diagnostic utility value.^10^ For each attribute, the levels were mean centered to zero with weighted effects coding to place the levels on a standard, linear scale. This process allows comparisons between and within the included attributes. A positive diagnostic utility value can be interpreted as having more diagnostic utility than the mean value of a given attribute. Negative diagnostic utility values indicate that the attribute level is below average in its diagnostic utility.

In addition to reporting the diagnostic utility value for each attribute level, we present the relative importance of each attribute. The relative importance was calculated using the maximum coefficient value minus the minimum coefficient value within each attribute, divided by the sum of these differences for all attributes.^10^ Higher percentage indicates greater importance of that attribute relative to other attributes assessed. Standard errors were clustered by respondent when calculating the 95% confidence intervals.^11^ These analyses were conducted separately for the 2 survey objectives; that is, values were calculated separately for influence of the selected attributes on ordering respiratory culture (all respondents) and influence on ordering BAL (among those choosing to order a respiratory culture).

## Results

### Participant characteristics

The electronic survey was sent to 300 eligible participants; 59 completed the survey in its entirety. Of these 59 HCP, 19 (32%) were attending physicians, 17 (29%) were clinical fellows, 12 (20%) were residents, 9 (16%) were advanced practice practitioners, and 2 (3.4%) were clinical pharmacists. The duration in clinical practice of respondents ranged from 3 years to >10 years, with 19 (32%) having >10 years of practice and 18 (31%) having 5–10 years of practice. Moreover, 28 participants (47%) identified the medical ICU as their primary place of work, 8 (14%) worked in the surgical ICU, 7 (12%) worked in the trauma ICU, 6 (10%) worked in the neuro ICU, and 2 (3.4%) worked in the cardiac ICU (Table 1).

**Table 1.** Characteristics of Respondents in a Discrete Choice Experiment Survey Among Critical Care Providers at an Academic Medical Center (N=59)

Characteristic	No. (%)^a^
**Clinical practice**	
ICU attending physician	19 (32)
Fellow	17 (29)
Resident	12 (20)
Nurse Practitioner	8 (14)
Clinical pharmacist	2 (3.4)
Physician assistant	1 (1.7)
**Years in practice**	
1–5 y	22 (37)
5–10 y	18 (31)
>10 y	19 (32)
**Clinical background**	
Pulmonary critical care	17 (29)
Surgical critical care medicine	17 (29)
Critical care medicine	10 (17)
Other	10 (17)
Neurology critical care medicine	5 (8.5)
**Primary place of work**	
Medical ICU	35 (59)
Surgical ICU	9 (15)
Trauma ICU	12 (20)
Neuro ICU	7 (12)
Cardiac ICU	3 (5%)
Cardiac surgery ICU	4 (7%)

a When summed, total percentage exceeds 100% because 11 respondents indicated >1 primary unit location.

### Decision to order respiratory cultures

**Fig. 2.**
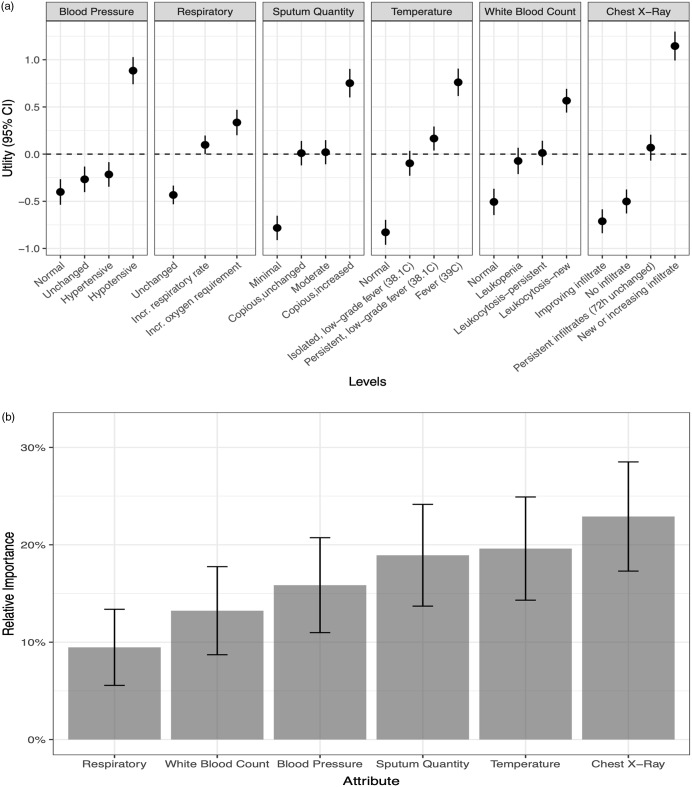
(a) The utility of individual levels of each clinical attribute for ventilator-associated pneumonia (VAP) evaluated in a discrete choice experiment among 59 critical care providers. Utility is reported on a linear scale with higher values associated with perceived greater diagnostic importance in the decision to order a respiratory culture for VAP diagnosis. Utility values are comparable across the attributes listed. (b) The relative importance of the 6 clinical attributes in decision to order a respiratory culture.

Among the 1,888 scenario responses, ordering a respiratory culture was selected in 911 plausible scenarios (48.3%). When evaluating individual attribute levels, new opacity on chest radiography had the strongest perceived diagnostic value, with a utility of 1.15 (95% CI, 0.99–1.3), followed by hypotension (utility, 0.88; 95% CI, 0.74–1.03), fever (utility, 0.76; 95% CI, 0.62–0.91), increasing copious sputum production (0.75; 95% CI, 0.60–0.90), new leukocytosis (0.57; 95% CI, 0.44–0.69) and increasing oxygen requirement (0.34; 95% CI, 0.2–0.47). Furthermore, the perceived diagnostic value of each attribute level can be appreciated relative to other levels of that attribute and can help identify which clinical findings are likely to influence an HCP to order a culture and which would dissuade an HCP from doing so (Fig. 2a). For example, a new chest radiograph infiltrate has utility of 1.15 (95% CI, 0.99–1.30) in comparision to no infiltrate (utility, −0.71; 95% CI, −0.84 to −0.59) or improving infiltrate (utility, −0.50; 95% CI, −0.63 to −0.38). Similarly, the presence of hypotension has utility of 0.88 (95% CI, 0.74–1.03) compared to normal blood pressure (utility, −0.40; 95% CI, −0.54 to −0.27), blood pressure unchanged from baseline (utility, −0.27; 95% CI, −0.40 to −0.13), or hypertensive patient (utility, −0.22; 95% CI, −0.83 to −0.09).

When evaluating the overall relative importance of clinical attributes (ie, comparing different attributes to one another in the decision to order respiratory cultures), changes in the patient’s chest radiograph (22.9%; 95% CI, 17.3%–28.5%), temperature (19.6%; 95% CI, 14.3%–24.9%), and sputum (18.9%; 95% CI, 13.7%–24.2%) had the greatest relative importance. Respiratory status changes (9.5%; 95% CI, 5.6%–13.4%) and changes in WBC (13.2%; 95% CI, 8.7%–17.8%) were relatively less important (Fig. 2b).

### Selecting bronchoscopy with BAL or mini-BAL

**Fig. 3.**
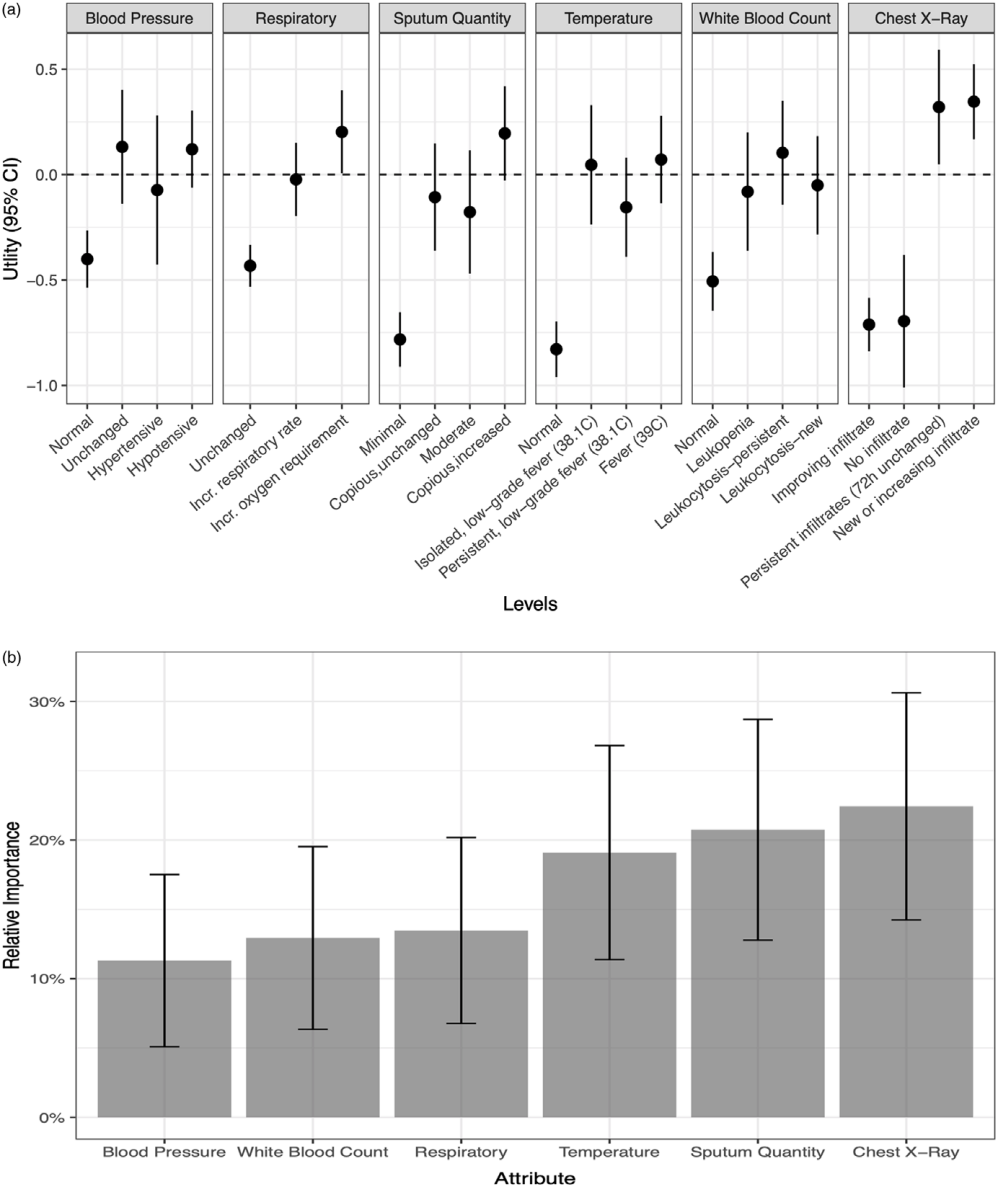
(a) The utility of individual levels of each clinical attribute for ventilator-associated pneumonia (VAP) evaluated in a discrete choice experiment among 59 critical care providers. Utility is reported on a linear scale with higher values associated with perceived greater diagnostic importance in the decision to select bronchoscopy with broncho-alveolar lavage (BAL)/mini-BAL over endotracheal aspirate when ordering a respiratory culture to diagnoseVAP. Utility values are comparable across the attributes listed. (b) The relative importance of the 6 clinical attributes in decision to select bronchoscopy with broncho-alveolar lavage (BAL)/mini-BAL over endotracheal aspirate.

Of 911 responses in which HCP chose to proceed with a respiratory culture, respondents preferred invasive sampling with BAL or mini-BAL over noninvasive sampling in 369 cases (40.5%). Among the individual attribute levels, observing new or increasing opacity on a chest radiograph (utility, 0.35; 95% CI, 0.17–0.52) and persistent opacity unchanged over the previous 72 hours (utility, 0.32; 95% CI, 0.05–0.59) were the 2 changes with the highest value toward selecting bronchoscopy with BAL or mini-BAL over noninvasive respiratory sampling (Fig. 3a). In contrast, having minimal sputum (utility, −0.78; 95% CI, −0.91 to −0.65), a normal temperature (utility, −0.83; 95% CI, −0.96 to −0.70), chest radiograph with no opacity (utility, −0.70; 95% CI, −1.01 to −0.38) or improving opacity (utility, −0.71; 95% CI, −0.84 to −0.58), and unchanged oxygenation (utility, −0.43; 95% CI, −0.53 to −0.33) discouraged HCPs from ordering a bronchoscopy with BAL or mini-BAL over noninvasive respiratory sampling.

When comparing the relative importance of these attributes in selecting bronchoscopy with BAL or mini-BAL (Fig. 3b), changes in chest radiograph had the greatest relative importance (22.4%; 95% CI, 14.2%–30.6%) compared to other clinical attributes. Sputum changes were second, with relative importance of 20.7% (95% CI, 12.8%–28.7%). Changes in WBC (11.3%; 95% CI, 5.1%–17.5%) were least important among the included attributes.

## Discussion

In our evaluation of 6 clinical attributes (ie, radiographic changes, respiratory and oxygenation status, sputum production, temperature, WBC, and blood pressure), both respiratory-specific and nonspecific symptoms had high clinical utility, as perceived by HCP, for pursuing respiratory tract culturing. Overall, chest imaging findings provided the most relative importance. Improving infiltrate strongly informed against ordering a respiratory culture, whereas new or increasing infiltrate strongly influenced the decision to order a respiratory culture. However, hypotension and fever had high diagnostic utility, similar to new opacity, suggesting that either of those two attributes can be important triggers for ordering a respiratory culture, even in the absence of additional respiratory specific symptoms.

Blood pressure as a whole had marginal relative importance in the decision to order a respiratory culture, given the respondents’ indifference toward normal, unchanged, and hypertensive readings. However, hypotension as an attribute level had high perceived diagnostic utility toward culturing for VAP diagnosis. Although the American Thoracic Society (ATS) and Infectious Diseases Society of America (IDSA) guidelines for VAP do not recognize hypotension as a qualified symptom for clinically defined pneumonia,^2^ changes in hemodynamics are heavily stressed for the detection of sepsis and to define septic shock.^12^ Our findings suggest that respiratory-tract culturing may be driven by sepsis vigilance.^6,13^ In a retrospective study by Kumar et al,^14^ the linear relationship outlining worsening mortality with delay in administration of antibiotics since onset of hypotension was instrumental in defining the management of sepsis. The surviving sepsis guideline has placed a strong emphasis on monitoring hemodynamics and promptly initiating antimicrobial therapy to improve outcomes in the critically ill.^12^ Although recognizing that early recognition of sepsis and antibiotics administration improves overall mortality,^12^ successfully differentiating infectious from noninfectious causes as contributory to worsening clinical status in the ICU remains challenging.^15^ Furthermore, both sepsis and antimicrobial stewardship guidelines emphasize obtaining cultures to guide antimicrobial therapy decisions. Unfortunately, those guidelines, when coupled with sepsis vigilance in a high-acuity population can drive indiscriminate “panculturing” in ICU patients in the absence of respiratory specific symptoms resulting in “false-positive” cultures. Such situations often lead to changes in antimicrobial selection but not discontinuation, even when site-specific criteria for infection are not met.

The perceived high diagnostic utility of fever similarly highlights a future target for diagnostic stewardship efforts. Fevers are common in critically ill patients, occurring in upward of 90% of those admitted to the ICU.^16,17^ The causes of fever in this patient population, however, are diverse and include a number of noninfectious etiologies.^18^ Fever in ICU patients often leads to reflexive panculturing, which contributes to VAP overdiagnosis and overtreatment.^19,20^ Such practice is even more pronounced in academic centers most cultures are ordered by trainees.^21^ A prospective survey of internal medicine residents showed that trainees practice panculturing as a reflexive response to fever knowing that it is not evidence based or cost-effective.^21^ Re-evaluation of the role of fever as a prompt for culturing, especially without consideration of focused, site-specific signs or symptoms, is needed to decrease overdiagnosis of infections such VAP.

Although the finding of new opacity is a driver for respiratory culturing overall, persistent chest radiograph opacity was identified as the most significant clinical finding for selecting bronchoscopy with BAL/mini-BAL over noninvasive sampling. This finding may be due to a perceived benefit of bronchoscopy and BAL in providing additional information beyond culture data, thus helping to differentiate infectious from noninfectious etiology.^4,22^ Advocates of lower respiratory culturing also highlight the benefit of site-specific sample collection for diagnostic accuracy.^6^ Furthermore, the resulting quantitative culture data could more reliably rule out infectious causes and could potentially contribute to early discontinuation of antibiotics.^23^

Our findings also highlight the ongoing debate regarding the validity and utility of changes in oxygenation as an important marker toward VAP clinical diagnosis and surveillance reporting.^24^ Both the ATS/IDSA guidelines and the CDC surveillance definitions place emphasis on worsening oxygenation as a defining attribute.^2,3^ However, in this study, respondents had variable to limited confidence in the diagnostic utility of increase in oxygenation requirement, which may reflect the perceived contribution from noninfectious causes such as volume overload, atelectasis, or acute respiratory distress syndrome.^4,22^ In contrast, respondents do appear to rely on the negative predictive value of lack of worsening oxygenation, as evidenced by its negative utility for ordering respiratory cultures. Similarly, improving chest radiograph, normal blood pressure, and normal temperature, all had negative utility for ordering respiratory cultures.

This study had several notable strengths and limitations. We used a survey that identified providers’ perceptions related to VAP diagnosis, but these findings may not reflect actual clinical practice, which could be different from providers’ perceptions of their actions in the hypothetical scenarios. Furthermore, each clinical attribute informs the perceivied diagnostic utility of that attribute and cannot be applied to inform the cumulative value of multiple combined attributes. Also, one-third of respondents were attending physicians, which may not reflect the practices of all levels of providers; however, ICU clinical decisions are often based on team rounds and discussions that are led by attending physicians. Finally, given the influence of local culture and practice norms, our findings may not be generalizable to other institutions. However, the application of this unique methodology to help define needs in diagnostic stewardship can be replicated. To our knowledge, this is the first study to utilize discrete choice experiment methodology to understand the importance of individual clinical attributes of VAP as drivers of respiratory culturing. When people are asked to rate an attribute, they often reply that “it depends” (on other accompanying factors); therefore, DCEs are considered superior to other survey methods for assessing preferences because they are able to measure these implicit trade-offs between attributes. Secondly, several studies have reported that DCE respondents complete these types of surveys in an internally valid and consistent manner.^25–27^

Clinician beliefs and cognitive characteristics play an important role in overdiagnosis and misdiagnosis in infectious diseases.^28,29^ The perceived high diagnostic importance of fever and hypotension suggests that sepsis vigilance may drive respiratory culturing, and as such, hypotension (though not part of VAP defining criteria) may play a role in VAP overdiagnosis. These findings can inform future work in diagnostic stewardship for VAP related to evaluating and addressing respiratory culturing occurring in the presence of fever, hypotension, or sepsis without accompanying respiratory changes.

## Supplementary material

For supplementary material accompanying this paper visit https://doi.org/10.1017/ash.2022.267. click here to view supplementary material
