# Supplementary material for: Understanding healthcare provider preferences for ordering respiratory cultures to diagnose ventilator associated pneumonia: A discrete choice experiment
Source: Antimicrob Steward Healthc Epidemiol. 2022 Jul 15;2(1):e120. doi: 10.1017/ash.2022.267 (PMC9726546; doi:10.1017/ash.2022.267)
Supplement: Supplementary file 1 [file S2732494X22002674sup001.pdf]

Dear colleague,

You are being asked to take part in this study because as a member of your professional group you are at the forefront of managing critically ill patients at risk for ventilator associated pneumonia.

The purpose of this study is to understand current practices for the diagnosis of ventilator associated pneumonia (VAP). We are particularly interested in learning how clinicians decide to initiate diagnostic work up for VAP based on presenting symptoms and signs.

Your role

We are conducting a survey of different members of the ICU team who are involved in the care of patients at risk for VAP. Participation will involve completing an anonymous online survey which should take no more than 15 minutes to complete

- Participation is VOLUNTARY, and you may choose not to participate at any time. Refusal to participate will not involve a penalty or loss of benefits to which you are otherwise entitled.

Risks

There is minimal risk to you as a participant

- Responses are collected anonymously and will not be linked back to the respondent.
- You do not have to answer any question that you choose not to answer.

Consent

By clicking the survey link below, you are providing consent to participate in this study.

***** If you decide to participate in this study, you will be enrolled in a raffle prize to win one of two $70 Amazon gift cards. If you wish to be entered in the raffle, please make sure to enter your email address after completing the survey (e-mail will not be linked to your responses).**

THANK YOU FOR YOUR HELP WITH THIS STUDY!

QUESTIONS
If you have any questions or concerns about this study, please contact:

Surbhi Leekha, MD
Associate Professor
Department of Epidemiology and Public Health
Department of Medicine
Division of Infectious disease
Tel: 410-328-1148
Email: sleekha@som.umaryland.edu

Blaine Kenaa, MD
Clinical Instructor
Department of Medicine
Division of Pulmonary and Critical Care Medicine
Tel: 240-472-3797
Email: blaine.kenaa@som.umaryland.edu

If not participating, please provide reason

[ ]Not enough time.

[ ]Not interested

[ ]This study has no relevance to me

[ ]Other ___________________

Demographic

Clinical role

[ ]ICU attending physician
[ ]Fellow
[ ]Resident
[ ]Clinical pharmacist
[ ]Nurse Practitioner
[ ]Physician assistant

Years in practice (including residency training)

A. 1-3 years   B. 3-5 years   C. 5-10 years   D. > 10 years

Clinical background

[ ]Pulmonary Critical Care
[ ]Critical Care Medicine
[ ]Surgical Critical Care Medicine
[ ]Anesthesia Critical Care Medicine
[ ]Neuro Critical Care Medicine

[ ]Other ____________________

Primary place of work (can check up to 2)

A. Medical ICU   B. Cardiac surgery ICU   C. Neuro ICU
D. Surgical ICU   E. Trauma ICU   F. Cardiac ICU

***** This survey is not intended to include any patients that have been diagnosed with COVID-19**

The questions below are all related to the same case vignette provided below. **We are asking your opinion on diagnostic approach to *respiratory tract bacterial culturing* based on different potential combinations of symptoms and signs developing in this patient**. Each question is to be answered as an independent clinical course on ICU day 7

> 54 y/o M with hypertension is admitted to the ICU 7 days prior with subarachnoid hemorrhage and managed without neurosurgical intervention. The patient remains on minimal ventilatory support, FiO2 40% with daily ventilator weaning assessment.

1. On your assessment today, this patient has:
-Isolated low- grade fever (38.1 C)
-Persistent opacity or infiltrates on chest x-ray, unchanged over the last 72 hours
-Increased Oxygen requirement
-Copious sputum production, increased from prior
-Leukopenia
-Hypotension

In this scenario, you would:
[ ] NOT send respiratory culture
[ ] Send respiratory culture

If Send respiratory culture, you would prefer:
[ ]Bronchoscopy with BAL/Mini-BAL
[ ]Endotracheal aspirate

2. On your assessment today, this patient has
-Normal temperature
-No infiltrate on chest x-ray
-Respiratory status unchanged
-Copious sputum production, increased from prior
-Leukocytosis, persistent but unchanged
-Blood pressure unchanged

In this scenario, you would:
[ ] NOT send respiratory culture
[ ] Send respiratory culture

If Send respiratory culture, you would prefer:
[ ]Bronchoscopy with BAL/Mini-BAL
[ ]Endotracheal aspirate

3. On your assessment today, the patient has: CU 2.006
-Fever of 39 C (0.761)
-New or increasing infiltrate/opacity on chest x-ray (1.145)
-Increased oxygen requirement (0.335)
-Moderate sputum production (0.020)
-Leukocytosis, persistent but unchanged (0.012)
-Blood pressure unchanged (-0.267)

In this scenario, you would:
[ ] NOT send respiratory culture
[ ] Send respiratory culture

If Send respiratory culture, you would prefer:
[ ]Bronchoscopy with BAL/Mini-BAL
[ ]Endotracheal aspirate

4. On your assessment today, the patient has:
-Isolated/single episode low-grade fever (38.1)
-Improving infiltrate/opacity on chest x-ray
-Respiratory status unchanged
-Copious sputum production, increased from prior
-Leukocytosis, new
-Blood pressure unchanged

In this scenario, you would:
[ ] NOT send respiratory culture
[ ] Send respiratory culture

If Send respiratory culture, you would prefer:
[ ]Bronchoscopy with BAL/Mini-BAL
[ ]Endotracheal aspirate

5. On your assessment today, the patient has
-Normal temperature
-Improving infiltrate/opacity on chest x-ray
-Increased oxygen requirement
-Minimal sputum production
-Normal white blood count
-Normal blood pressure

In this scenario, you would:
[ ] NOT send respiratory culture
[ ] Send respiratory culture

If Send respiratory culture, you would prefer:
[ ]Bronchoscopy with BAL/Mini-BAL
[ ]Endotracheal aspirate

6. On your assessment today, the patient has:
-Isolated/ single episode low-grade fever (38.1)

-New or increasing infiltrate/opacity on chest x-ray
-Increased respiratory rate
-Moderate sputum production
-Leukocytosis, persistent but unchanged
-Normal blood pressure

In this scenario, you would:
[ ] NOT send respiratory culture
[ ] Send respiratory culture

If Send respiratory culture, you would prefer:
[ ]Bronchoscopy with BAL/Mini-BAL
[ ]Endotracheal aspirate

7. On your assessment today, the patient has:
-Fever 39 C
-Persistent opacity or infiltrates on chest x-ray, unchanged over last 72 hours
-Increased respiratory rate
-Copious sputum, unchanged over several days
-Leukocytosis, new
-Unchanged blood pressure

In this scenario, you would:
[ ] NOT send respiratory culture
[ ] Send respiratory culture

If Send respiratory culture, you would prefer:
[ ]Bronchoscopy with BAL/Mini-BAL
[ ]Endotracheal aspirate

8. On your assessment today, the patient has:
-Fever 39 C
-No infiltrate on chest x-ray
-Increased respiratory rate

-Minimal sputum
-Leukopenia
-Hypertensive

In this scenario, you would:
[ ] NOT send respiratory culture
[ ] Send respiratory culture

If Send respiratory culture, you would prefer:
[ ]Bronchoscopy with BAL/Mini-BAL
[ ]Endotracheal aspirate

9. On your assessment today, the patient has
-Normal Temperature
-No infiltrate on chest x-ray
-Increased respiratory rate
-Moderate sputum production
-Leukocytosis, new
-Hypotensive

In this scenario, you would:
[ ] NOT send respiratory culture
[ ] Send respiratory culture

If Send respiratory culture, you would prefer:
[ ]Bronchoscopy with BAL/Mini-BAL
[ ]Endotracheal aspirate

10. On your assessment today, the patient has:
-Isolated/single episode low-grade fever (38.1 C)
-Persistent opacity or infiltrates on chest x-ray, unchanged over last 72 hours
-Increased oxygen requirement
-Moderate sputum production
-Leukocytosis, new

-Hypertensive

In this scenario, you would:
[ ] NOT send respiratory culture
[ ] Send respiratory culture

If Send respiratory culture, you would prefer:
[ ]Bronchoscopy with BAL/Mini-BAL
[ ]Endotracheal aspirate

11. On your assessment today, the patient has:
-Fever 39 C
-Improving infiltrate/opacity on chest x-ray
-Increased oxygen requirement
-Minimal sputum production
-Leukocytosis, new
-Hypotensive

In this scenario, you would:
[ ] NOT send respiratory culture
[ ] Send respiratory culture

If Send respiratory culture, you would prefer:
[ ]Bronchoscopy with BAL/Mini-BAL
[ ]Endotracheal aspirate

12. On your assessment today, the patient has:
-Persistent low-grade fever (38.1 C)
-New or increasing infiltrate/opacity on chest x-ray
-Increased oxygen requirement
-Copious sputum, increased from prior
-Leukopenia
-Hypertensive

In this scenario, you would:
[ ] NOT send respiratory culture
[ ] Send respiratory culture

If Send respiratory culture, you would prefer:
[ ]Bronchoscopy with BAL/Mini-BAL
[ ]Endotracheal aspirate

13. On your assessment today, the patient has:
-Normal Temperature
-New or increasing infiltrate/opacity on chest x-ray
-Increased oxygen requirement
-Copious sputum, unchanged
-Leukocytosis, new
-Normal blood pressure

In this scenario, you would:
[ ] NOT send respiratory culture
[ ] Send respiratory culture

If Send respiratory culture, you would prefer:
[ ]Bronchoscopy with BAL/Mini-BAL
[ ]Endotracheal aspirate

14. On your assessment today, the patient has:
-Fever (39 C)
-No infiltrate on chest x-ray
-Oxygenation unchanged from baseline
-Moderate sputum production
-Leukopenia
-Normal

In this scenario, you would:
[ ] NOT send respiratory culture
[ ] Send respiratory culture

If Send respiratory culture, you would prefer:
[ ]Bronchoscopy with BAL/Mini-BAL
[ ]Endotracheal aspirate

15. On your assessment today, the patient has:
-Fever (39 C)
-Persistent opacity or infiltrates on chest x-ray, unchanged over last 72 hours
-Oxygenation unchanged from baseline
-Copious sputum, increased from prior
-Normal white blood count
-Normal blood pressure

In this scenario, you would:
[ ] NOT send respiratory culture
[ ] Send respiratory culture

If Send respiratory culture, you would prefer:
[ ]Bronchoscopy with BAL/Mini-BAL
[ ]Endotracheal aspirate

16. On your assessment today, the patient has:
-Fever of (39 C)
-New or increasing infiltrate/opacity on chest x-ray
-Increased respiratory rate
-Copious sputum, increased from prior
-Normal white blood count
-Hypotensive

In this scenario, you would:
[ ] NOT send respiratory culture
[ ] Send respiratory culture

If Send respiratory culture, you would prefer:
[ ]Bronchoscopy with BAL/Mini-BAL
[ ]Endotracheal aspirate

17. On your assessment today, the patient has:
-Normal Temperature
-New or increasing infiltrate/opacity on chest x-ray
-Oxygenation unchanged from baseline
-Copious sputum, unchanged over several days
-Leukopenia
-Hypotensive

In this scenario, you would:
[ ] NOT send respiratory culture
[ ] Send respiratory culture

If Send respiratory culture, you would prefer:
[ ]Bronchoscopy with BAL/Mini-BAL
[ ]Endotracheal aspirate

18. On your assessment today, the patient has:
-Normal Temperature
-Persistent opacity or infiltrate on chest x-ray, unchanged over last 72 hours
-Oxygenation unchanged from baseline
-Minimal sputum production
-Leukopenia
-Blood pressure unchanged

In this scenario, you would:
[ ] NOT send respiratory culture
[ ] Send respiratory culture

If Send respiratory culture, you would prefer:
[ ]Bronchoscopy with BAL/Mini-BAL
[ ]Endotracheal aspirate

19. On your assessment today, the patient has:
-Persistent low-grade fever (38.1 C)
-No infiltrate on chest x-ray
-Increased oxygen requirement
-Copious sputum, unchanged over several days
-Normal white blood count
-Unchanged blood pressure

In this scenario, you would:
[ ] NOT send respiratory culture
[ ] Send respiratory culture

If Send respiratory culture, you would prefer:
[ ]Bronchoscopy with BAL/Mini-BAL
[ ]Endotracheal aspirate

20. On your assessment today, the patient has:
-Isolated/single episode low-grade fever (38.1 C)
-New or increasing infiltrate/opacity on chest x-ray
-Increased respiratory rate
-Minimal sputum production
-Normal white Blood count
-Unchanged blood pressure

In this scenario, you would:
[ ] NOT send respiratory culture
[ ] Send respiratory culture

If Send respiratory culture, you would prefer:
[ ]Bronchoscopy with BAL/Mini-BAL

[ ]Endotracheal aspirate

21. On your assessment today, the patient has:
-Persistent low-grade fever (38.1C)
-New or increasing infiltrate/opacity on chest x-ray
-Oxygenation unchanged from baseline
-Minimal sputum production
-Leukocytosis, new
-Hypertensive

In this scenario, you would:
[ ] NOT send respiratory culture
[ ] Send respiratory culture

If Send respiratory culture, you would prefer:
[ ]Bronchoscopy with BAL/Mini-BAL
[ ]Endotracheal aspirate

22. On your assessment today, the patient has:
-Isolated/single episode low-grade fever (38.1 C)
-No infiltrate on chest x-ray
-Oxygenation unchanged from baseline
-Copious sputum production, unchanged over several days
-Normal white Blood count
-Hypertensive

In this scenario, you would:
[ ] NOT send respiratory culture
[ ] Send respiratory culture

If Send respiratory culture, you would prefer:
[ ]Bronchoscopy with BAL/Mini-BAL
[ ]Endotracheal aspirate

23. On your assessment today, the patient has:
-Isolated/single episode low-grade fever (38.1 C)
-No infiltrate on chest x-ray (-0.502)
-Increased oxygen requirement (0.335)
-Minimal sputum production (-0.782)
-Leukocytosis, persistent but unchanged (0.012)
-Hypotensive (0.884)

In this scenario, you would:
[ ] NOT send respiratory culture
[ ] Send respiratory culture

If Send respiratory culture, you would prefer:
[ ]Bronchoscopy with BAL/Mini-BAL
[ ]Endotracheal aspirate

24. On your assessment today, the patient has:
-Persistent low-grade fever (38.1 C)
-Persistent opacity or infiltrates on chest x-ray, unchanged over last 72 hours
-Increased respiratory rate
-Copious sputum production, unchanged over several days
-Leukocytosis, persistent but unchanged
-Hypotensive

In this scenario, you would:
[ ] NOT send respiratory culture
[ ] Send respiratory culture

If Send respiratory culture, you would prefer:
[ ]Bronchoscopy with BAL/Mini-BAL
[ ]Endotracheal aspirate

25. On your assessment today, the patient has:
-Isolated/single episode low-grade fever (38.1 C)

-Improving infiltrate/opacity on chest x-ray
-Increased respiratory rate
-Copious sputum production, unchanged over several days
-Leukopenia
-Normal Blood pressure

In this scenario, you would:
[ ] NOT send respiratory culture
[ ] Send respiratory culture

If Send respiratory culture, you would prefer:
[ ]Bronchoscopy with BAL/Mini-BAL
[ ]Endotracheal aspirate

26. On your assessment today, the patient has:
-Persistent low-grade fever
-Improving infiltrate/opacity on chest x-ray
-Oxygenation unchanged from baseline
-Moderate sputum production
-Normal white blood count
-Hypotensive

In this scenario, you would:
[ ] NOT send respiratory culture
[ ] Send respiratory culture

If Send respiratory culture, you would prefer:
[ ]Bronchoscopy with BAL/Mini-BAL
[ ]Endotracheal aspirate

27. On your assessment today, the patient has:
-Normal temperature
-Improving infiltrate/opacity on chest x-ray
-Increased respiratory rate

-Copious sputum production, increased from prior
-Leukocytosis, persistent but unchanged
-Hypertensive

In this scenario, you would:
[ ] NOT send respiratory culture
[ ] Send respiratory culture

If Send respiratory culture, you would prefer:
[ ]Bronchoscopy with BAL/Mini-BAL
[ ]Endotracheal aspirate

28. On your assessment today, the patient has:
-Persistent low-grade fever (38.1 C)
-No infiltrate on chest x-ray
-Increased respiratory rate
-Copious sputum production, increased from prior
-Leukocytosis, new
-Normal blood pressure

In this scenario, you would:
[ ] NOT send respiratory culture
[ ] Send respiratory culture

If Send respiratory culture, you would prefer:
[ ]Bronchoscopy with BAL/Mini-BAL
[ ]Endotracheal aspirate

29. On your assessment today, the patient has:
-Normal temperature
-Persistent opacity or infiltrates on chest x-ray, unchanged over last 72 hours
-Increased respiratory rate
-Moderate amount sputum production
-Normal white blood count
-Hypertensive

In this scenario, you would:
[ ] NOT send respiratory culture
[ ] Send respiratory culture

If Send respiratory culture, you would prefer:
[ ]Bronchoscopy with BAL/Mini-BAL
[ ]Endotracheal aspirate

30. On your assessment today, the patient has:
-Fever (39 C)
-Improving infiltrate/opacity on chest x-ray
-Oxygenation unchanged from baseline
-Copious sputum production, unchanged over several days
-Leukocytosis, persistent but unchanged
-Hypertensive

In this scenario, you would:
[ ] NOT send respiratory culture
[ ] Send respiratory culture

If Send respiratory culture, you would prefer:
[ ]Bronchoscopy with BAL/Mini-BAL
[ ]Endotracheal aspirate

31. On your assessment today, the patient has:
-Persistent low-grade fever (38.1 C)
-Persistent opacity of infiltrates on chest x-ray, unchanged over last 72 hours
-Oxygenation unchanged from baseline
-Minimal sputum production
-Leukocytosis, persistent but unchanged
-Normal blood pressure

In this scenario, you would:

[ ] NOT send respiratory culture
[ ] Send respiratory culture

If Send respiratory culture, you would prefer:
[ ]Bronchoscopy with BAL/Mini-BAL
[ ]Endotracheal aspirate

32. On your assessment today, the patient has:
-Persistent low-grade fever (38.1 C)
-Improving infiltrate/opacity on chest x-ray
-Increased respiratory rate
-Moderate sputum production
-Leukopenia
-Unchanged blood pressure

In this scenario, you would:
[ ] NOT send respiratory culture
[ ] Send respiratory culture

If Send respiratory culture, you would prefer:
[ ]Bronchoscopy with BAL/Mini-BAL
[ ]Endotracheal aspirate
